# The Multifaceted Roles of USP15 in Signal Transduction

**DOI:** 10.3390/ijms22094728

**Published:** 2021-04-29

**Authors:** Tanuza Das, Eun Joo Song, Eunice EunKyeong Kim

**Affiliations:** 1 Biomedical Research Institute, Korea Institute of Science and Technology, Seoul 02793, Korea; tanuza.bge@gmail.com; 2 Graduate School of Pharmaceutical Sciences, College of Pharmacy, Ewha Womans University, Seoul 03760, Korea

**Keywords:** USP15, TGF-β, BMP, NF-κB, Wnt/β-catenin, CSN, p53, IGF, Nrf2–Keap1, RNA splicing

## Abstract

Ubiquitination and deubiquitination are protein post-translational modification processes that have been recognized as crucial mediators of many complex cellular networks, including maintaining ubiquitin homeostasis, controlling protein stability, and regulating several signaling pathways. Therefore, some of the enzymes involved in ubiquitination and deubiquitination, particularly E3 ligases and deubiquitinases, have attracted attention for drug discovery. Here, we review recent findings on USP15, one of the deubiquitinases, which regulates diverse signaling pathways by deubiquitinating vital target proteins. Even though several basic previous studies have uncovered the versatile roles of USP15 in different signaling networks, those have not yet been systematically and specifically reviewed, which can provide important information about possible disease markers and clinical applications. This review will provide a comprehensive overview of our current understanding of the regulatory mechanisms of USP15 on different signaling pathways for which dynamic reverse ubiquitination is a key regulator.

## 1. Introduction

Ubiquitination is a post-translational modification (PTM) process involving the covalent attachment of 76-amino acid ubiquitin to the target proteins through the sequential activation of the ubiquitin-activating enzyme (E1), ubiquitin-conjugating enzyme (E2), and ubiquitin ligase (E3) [1]. Ubiquitin modification regulates cellular functions by triggering protein degradation via the proteasomal or lysosomal pathway, by activating or inactivating target proteins, by redirecting the cellular localization of specific proteins, or by modifying the protein–protein interaction status in cells [2,3]. Deubiquitination is the reverse process of ubiquitination, which is mediated by a group of proteases known as deubiquitinating enzymes (DUBs) [4]. Nearly 100 putative DUBs encoded by the human genome have been identified so far. They are classified into seven families based on their catalytic domain features and functional similarities, namely, the ubiquitin-specific proteases (USPs), ubiquitin C-terminal hydrolases (UCHs), ovarian tumor proteases (OTUs), Machado–Joseph disease proteases (MJDs), motif interacting with ubiquitin-containing novel DUB family members (MINDYs), zinc finger-containing ubiquitin peptidase 1 (ZUP1), and JAB1/MPN/Mov34 proteases (JAMMs) [5,6,7,8]. Among the different groups, the USPs are the most numerous and versatile class of DUBs, consisting of ~70 members in humans [9], with their protein molecular weight ranging from 50 to 300 kDa [10]. In all of the USPs, the catalytic site contains a cysteine residue with nearby histidine and asparagine/aspartate residues that assist the cysteine in the nucleophilic attack. [11]. Because of the large number of substrates identified to date, USPs play versatile roles in different cellular processes, such as protein degradation, chromatin remodeling, DNA repair, cell cycle regulation, metastasis, endocytosis, and regulation of signal transduction. Despite belonging to the same group, individual members of the USP family show high functional diversity in different cellular pathways owing to their multiple regulatory mechanisms in cells.

USP15 (ubiquitin-specific peptidase 15 or ubiquitin carboxyl-terminal hydrolase 15), a member of the USP family, is widely expressed in different tissues and regulates numerous cellular processes [12]. To date, several substrates of USP15 have been identified, and their functions and regulatory roles have been well studied. In particular, USP15 draws specific attention because of the conflicting functions of its substrates in cancer biology, which raises the question of whether USP15 is more likely to act as a tumor suppressor or as an oncogene. There is evidence of copy number gains of the *USP15* gene in glioblastoma and breast and ovarian cancers [13], whereas copy number losses have been reported for *USP15* in pancreatic cancer [14]. Although there is a report revealing that USP15 may be associated with the 26S proteasome during substrate degradation [15], the functional details need further study. Many of the proposed substrates of USP15 in different cancer-associated pathways are involved in several signaling cascades. Here, we will provide a comprehensive overview of USP15 regulatory mechanisms in different cellular signaling pathways, which may help us to gain a better understanding of the biological functions of USP15 and guide future research for the development of suitable USP15 targeting therapeutic systems.

## 2. USP15 Gene and Its Paralogs

Human *USP15* mRNA is ubiquitously found in various tissues and organs, most abundantly in the pancreas, testes, thyroid, and adrenal gland based on its observed expression in the mouse and that of the rat orthologue *Ubp109* [16]. The *USP15* gene is located on chromosome 12q14.1, spanning 149,382 bases of genomic DNA. Nineteen different splicing variants are found to be encoded by the *USP15* gene, eight of which compose the coding region, while only three transcripts have the full-length exon structure. Four isoforms, with 981, 957, 952, and 235 amino acid residues, have been identified, and isoform-specific binding proteins, as well as PTM, have been reported, suggesting the isoform-specific role of USP15 in distinct cellular networks with overlapping but independent functions. For example, only isoform 1 is shown to recognize and deubiquitylate the moyamoya disease-associated ubiquitin ligase mysterin [17], and phosphorylation at residue Ser229 of isoform 1 is shown to regulate topoisomerase IIα to maintain genome integrity [12]. In isoforms 2 and 3, Ser229 is missing, while it is Lys in isoform 4.

The catalytic domain of USP15 has a canonical USP fold, including a finger, palm, and thumb region [18]. However, the crystal structure of the catalytic core domain of USP15 revealed the catalytic cysteine about 10 Å from the catalytic histidine, thereby requiring a realignment of the catalytic triads prior to the catalysis, as seen in a few USPs. In addition to the catalytic domain, USP15 has an N-terminal DUSP domain and the ubiquitin-like (UBL) domain. The DUSP domain, consisting of approximately 90 amino acid residues, forms a tripodlike structure with a three-α-helix bundle supporting a three-stranded antiparallel β-sheet resembling the legs and seat of a tripod, while the UBL domain, consisting of about 80 residues, forms the β-grasp fold [19,20,21]. The function of the DUSP domain is currently unknown, although it has been suggested that it may play a role in protein–protein interactions, in particular for DUB substrate recognition. However, in the case of USP15, the DUSP domain, together with the UBL domain, plays a critical role in the interaction with squamous cell carcinoma antigen recognized by T-cells 3 (SART3), allowing it to carry out its function in spliceosome recycling [22,23,24]. A recent study showed that the differential phosphorylation of USP15 on the UBL region (Thr149 and Thr219) results in an altered interaction with SART3 that affects USP15 localization and activity toward its substrate [25].

The same overall structural organization of USP15, an N-terminal DUSP–UBL domain followed by the catalytic domain, is found in USP4 and USP11 with sequence similarities of 57% and 43%, respectively [26,27,28]. In all three paralogs, the protease domain harbors a ~300 amino acid insertion predicted to contain a second UBL domain. These ancillary domains affect the catalytic function in different ways by regulating the catalytic activity of USP4 while USP11′s activity is not significantly modulated [18,29]. The enzyme kinetics of USP15 and USP4 are more similar to each other than those of USP11, even though the catalytic residues at the active site of USP4 are in their canonical positions unlike in USP15 [29]. In the case of USP4, the N-terminal DUSP–UBL regulates the DUB activity in the spliceosome as in USP15; that is, the highly conserved linker region with residues ^125^GLFVKH^130^ between the DUSP and UBL domains is essential for its interaction with SART3 and thereby for its nuclear shuttling [22]. Phosphorylation of the UBL region of USP4 shows a similar effect on SART3 interaction, localization, and substrate targeting to regulate the spliceosome dynamics [25]. However, in the case of USP11, such a regulatory mechanism is absent [21,29]. Evolutionary analysis revealed that both USP15 and USP4 are present in all organisms, while USP11 has been lost multiple times throughout the vertebrate evolution [26]. They show partially redundant functions in vital cellular pathways, such as cell development, proliferation, innate immunity, and several signaling networks. In particular, the elevated expression of these three paralogs has been reported in several human malignancies, which, in turn, modulates the functions of several tumor suppressors and oncogenes. To some extent, they are reported to be involved in different cancer-associated signal transduction pathways, such as TGF-β, including many of its downstream effectors [13,30,31]. Although these three paralogs are functionally redundant in numerous cellular aspects, each appears to have its specific targets and functions with distinctive signature features [26]. Several previous basic studies have revealed the versatile roles of USP4, USP11, and USP15 in different physiological and pathological processes, particularly in cancers. Recently, USP4 has been systematically and precisely reviewed with a good understanding of its structure, functions, and regulation [25,29]; however, USP15 seems to have drawn comparatively low attention. Consequently, the clinical application value of USP15 as a potential cancer target remains underestimated. Hence, this review will provide a comprehensive overview of the regulatory mechanisms of USP15, particularly in different cancer-associated signaling pathways, which might raise its potentiality as a target for cancer therapy and other clinical applications.

## 3. USP15 Regulates Signal Transduction Pathways

Cellular signal transduction is the process of transmitting chemical or physical signals by triggering a series of molecular events in the cell until the appropriate genes are expressed to trigger the biological effects in the form of cellular responses, such as cell growth, proliferation, and metabolism [32]. There are numerous signal transduction pathways in cells that are cross-regulated at multiple levels, resulting in a very complicated network system. Although protein phosphorylation and dephosphorylation, respectively catalyzed by kinases and phosphatases, play central roles in signal transduction, numerous other PTMs also have vital effects in different signaling networks, among which deubiquitination by USP15 has been revealed as an important regulatory mechanism. USP15 is reported to be involved in several signaling pathways, including TGF-β [13], nuclear factor kappa B (NF-ĸB) [33], p53 [34], β-catenin [35], neuroinflammation [36], T-cell activation [37], the Nrf2–Keap1 pathway [38], bone morphogenetic protein (BMP) signaling [39], and regulation of the COP9 signalosome (CSN) [35]. The functions of several other proteins involved in these signaling pathways may also be mediated by USP15. The following sections will discuss the regulatory mechanisms of USP15 in different signaling networks.

### 3.1. USP15 Enhances TGF-β Signaling by Deubiquitinating the Type I TGFβ Receptor ALK5

The TGF-β signaling pathway is involved in diverse cellular processes in both the developing embryo and the adult organism; these processes include cell growth and differentiation, immune response, apoptosis, cellular homeostasis, wound healing, and many other functions. This pathway is initiated by the binding of the secreted cytokine TGF-β or other ligands of this superfamily to the Ser/Thr receptor kinase TGF-β type II receptor, which recruits, phosphorylates, and activates the signal-transducing type I receptor. Intracellular signals are then transmitted through the phosphorylation of receptor-regulated SMADs (R-SMADs), which bind to the co-operating SMADs (coSMADs) and accumulate in the nucleus to switch on target gene expression [40,41]. Although the TGF-β signaling pathway is tightly regulated by phosphorylation, its activity is also regulated by reversible ubiquitination [42]. Several TGF-β pathway components, including receptors, R-SMADs, and coSMADs, are ubiquitinated by different E3 ubiquitin ligases [43]. As ubiquitination is reversed by deubiquitination, studies on DUBs regulating TGF-β signaling have focused on uncovering the key regulatory mechanism of DUBs in ubiquitin removal from the signaling components [44].

**Figure 1:**
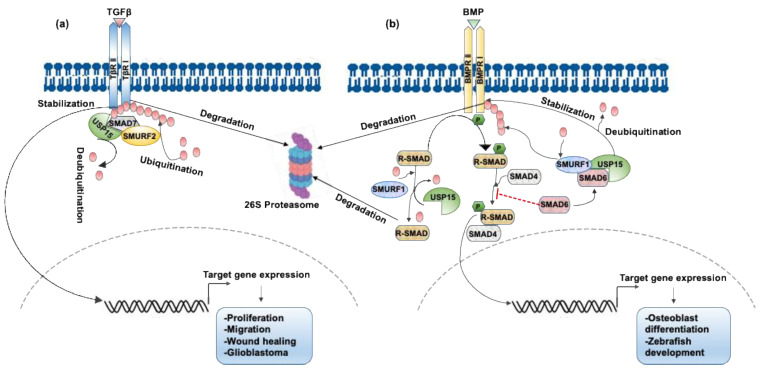
USP15 in the regulation of TGF-β and BMP signaling. (**a**) USP15 binds to SMAD7 and opposes SMURF2-mediated ubiquitination of TβRI. The deubiquitination and stabilization of TβRI by USP15 promotes TGF-β activity and triggers the downstream gene expression to stimulate cell proliferation, migration wound healing, and glioblastoma pathogenesis [13]. (**b**) Upon activation by BMP ligands, the receptor-regulated R-SMADs undergo phosphorylation, bind to SMAD4, and enter the nucleus to induce target gene transcription. SMAD6 is a negative regulator that competes with SMAD4 to bind to R-SMADs and induces SMURF1-mediated ubiquitination and degradation of R-SMADs. Through its interaction with SMAD6, USP15 deubiquitinates and stabilizes BMPR1 and R-SMADs to enhance the BMP signaling and elicit target gene expression [39].

RNAi-mediated loss of function of DUB screening libraries identified USP15 as a critical regulator of the TGF-β signaling pathway. USP15 stabilizes the TGF-β receptor as well as its downstream signal transducers, the R-SMADs, and enhances TGF-β activity. For example, the binding of USP15 to the SMAD7-specific E3 ubiquitin ligase 2 (SMURF2) complex results in the deubiquitination and stabilization of TGF-β receptor 1 (TβRI) and promotes TGF-β activity. USP15 forms a complex with SMAD7 and SMURF2 and opposes the SMURF2-mediated ubiquitination of TβRI when the level of active TGF-β is low [13]. Apart from directly targeting the TβRI complex, USP15 also deubiquitinates the E3 ligase SMURF2, leading to enhanced TβR stability and activation of its downstream pathways. Here, USP15 modulates Lys734 ubiquitination within the C-lobe of SMURF2, which is required for its catalytic activity [45]. The inclusive regulation of TGF-β signaling by USP15 is illustrated in Figure 1a. The *USP15* gene is highly amplified in glioblastoma and ovarian and breast cancers, and this high expression correlates with enhanced TGF-β activity. In contrast, the depletion of USP15 in a glioblastoma orthotopic mouse model decreases TGF-β activity. High levels of amplification of *USP15* in individuals with glioblastoma are associated with poor prognosis [13]. Additionally, USP15 shows a significant positive correlation with SMAD7 and a substantial negative correlation with TβRI expression in the systemic skin inflammatory disease psoriasis, although the concrete mechanism behind this regulation remains unclear [46]. TGF-β signaling may either enhance aggressive cell migration or induce growth arrest, depending on the particular cellular context [47], where USP15 plays a critical role in both types of responses. In immortalized HaCaT keratinocytes, *USP15* knockdown impairs TGF-β/SMAD-dependent growth arrest but is required for TGF-β-induced cell motility in metastatic MDA-MB-231 breast cancer cells [48]. Overexpression of *USP15* is also found to increase the response to TGF-β in systemic sclerosis fibroblasts [49]. Additionally, a recent report showed that USP15 deubiquitinates TβRI to induce wound healing by promoting the proliferation and migration of human dermal fibroblasts, thus proving to be a new potential target to treat refractory wounds [50].

It is not surprising that USP4 and USP11 can also potentiate TGF-β signaling, as they share high sequence similarity and conserved domains with USP15. However, whether these three very closely related DUBs work in conjunction with each other or act independently is still under investigation. Published studies revealed that, unlike USP15, USP4 directly binds, stabilizes, and activates TβRI in the plasma membrane rather than via SMAD7-mediated recruitment to the active receptor as USP15 does. USP4 is associated with and phosphorylated by AKT at its conserved Ser445 residue that promotes membrane and cytoplasmic localization of USP4, where it deubiquitinates TβRI [31]. In vitro and in vivo studies also propose USP4 as a selective regulator of TGF-β/SMAD signaling in mammalian cells and zebrafish embryos that plays a critical role in the tumor-inducing arm of the TGF-β/SMAD pathway [51,52]. Likewise, a systematic proteomic approach identified USP11 as a novel binding partner of TGF-β signaling components and a critical regulator of TGF-β signaling. USP11 promotes TGF-β signaling by deubiquitinating the TβRI ALK5 through interaction with and recruitment via SMAD7 [30]. Therefore, USP15, USP4, and USP11 show strong functional links to the TGF-β/SMAD pathway and thus regulate TGF-β-induced epithelial–mesenchymal transition, migration, invasion, metastasis, and several other related functions.

### 3.2. USP15 Deubiquitinates Type I BMP Receptor ALK3 and Enhances BMP Signaling

In addition to TGF-β signaling, USP15 is necessary for the regulation of other TGF-β superfamily members, such as the bone morphogenetic protein (BMP), in mammalian cells and *Xenopus* embryos, as shown in Figure 1b. USP15 interacts with a negative regulator of BMP signaling, SMAD6, which enhances SMURF1/2-mediated ubiquitination and proteasomal degradation of the type I BMP receptor ALK3. USP15 deubiquitinates ALK3 through its interaction and colocalization with ALK3 at the plasma membrane and thereby opposes SMAD6-mediated inhibition of BMP signaling. The depletion of USP15 increases ALK3 K48-linked polyubiquitination and decreases BMP-induced SMAD1 phosphorylation and BMP target gene transcription. Thus, USP15 is a key regulator of the BMP signaling pathway in *Xenopus* embryos and in human and mouse cells via its promotion of BMP-induced SMAD1 phosphorylation, target gene transcription, and osteoblast differentiation [39]. Besides ALK3, USP15 also interacts with and deubiquitinates monoubiquitinated R-SMADs, causing enhanced TGF-β and BMP responses in both mammalian cells and *Xenopus* embryos. In this pathway, USP15 inhibits R-SMAD monoubiquitination, which targets R-SMAD’s DNA-binding domain and prevents promoter recognition, resulting in increased TGF-β and BMP signaling [48]. Additionally, USP15 mediates BMP-dependent dorsoventral patterning by the zebrafish TGF-β-stimulated clone 22 (TSC22D3), which is reported to exert a positive effect on the BMP signaling pathway by stimulating the transcription of BMP4 [53]. A genomewide loss-of-function study of DUBs also suggested that they play an important role in BMP signaling that affects dorsoventral patterning during zebrafish development [54]. Nevertheless, the other two related DUBs—USP4 and USP11—have not yet shown any significant effects on the regulation of the BMP signaling pathway [30,31,39].

### 3.3. USP15 Regulates the Activity of the COP9 Signalosome toward CRLs

The COP9 signalosome (CSN) represents a multi-DUB complex with isopeptidase activity consisting of the conserved eight-subunit (CSN1–8) protein involved in the ubiquitin–proteasome pathway found in nearly all eukaryotic cells. CSN predominantly regulates the neddylation status of cells by destabilizing deneddylation enzyme 1 (DEN1). Additionally, CSN regulates the activity and assembly of cullin–RING ubiquitin ligases (CRLs) [55]. Thus, CSN acts as a master regulator at the intersection of neddylation and ubiquitination in the proteasomal degradation system. In addition to its association with deubiquitinating enzymes, CSN protects CRLs from autoubiquitination and rescues them from proteasomal degradation. This secure coordination of ubiquitination and deubiquitination by the CSN is critical for the fine-tuning of many important cellular pathways, such as the cell cycle, DNA damage, and apoptosis; the functional dysregulation of CSN, CRLs, and the corresponding E3 substrates gives rise to several diseases, including cancer [56,57].

To date, USP15 is the most well-characterized DUB among the few reported CSN-associated DUBs. Systematic mass spectrometric screening identified USP15, corresponding to Ubp12p in *Schizosaccharomyces pombe* and UspA in *Aspergillus nidulans* via its interaction with CSN, which is conserved among species. In *S. pombe*, the association of Ubp12p with CSN is required for its efficient nuclear transport to regulate the ubiquitination and stability of CRLs. In this process, CSN mediates the interaction of cullin and Ubp12p. This neutralizes cullin ubiquitin ligase activity by targeting Ubp12p to cullins and by maintaining the stability of cullin adaptor proteins [58]. In *A. nidulans*, UspA predominantly interacts with the helical bundle of the CSN that regulates fungal multicellular development and secondary metabolism [59]. However, in human cells, the interaction of CSN with USP15 is mediated by diverse mechanisms, but the specific subunits or variants involved in this interaction are still unknown. Evidence indicates that the functional Zn finger domain of USP15 is required to reverse the poly- or autoubiquitination of the cullin–RING E3 ligase Rbx1 by maintaining a conformation necessary for the disassembly of the poly-Ub chains [60]. By associating with the CSN, USP15 efficiently delivers the deubiquitination activity toward the CRL substrates to protect them from degradation by the proteasome. Besides protecting the CRL components during the remodeling process of the E3 ligase complexes, USP15 also negatively regulates the CRL substrates in the NF-ĸB and Wnt signaling pathways in conjunction with CSN, which will be briefly discussed in the later sections. To date, only two CSN-associated DUBs, USP15 and USP48, have been characterized [61,62]. Although additional CSN-associated DUBs have been identified through mass spectrometric analysis [63], further verification by appropriate methods is required to validate their association and the related functions. Nevertheless, there is no evidence of an association of CSN with USP4 or USP11.

### 3.4. USP15 Can Either Suppress or Activate NF-κB Signaling

**Figure 2:**
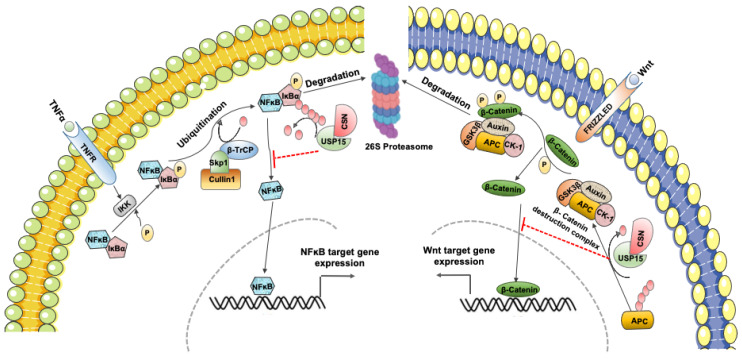
USP15 suppresses TNFα and Wnt signaling in association with CSN. Upon stimulation by TNFα, NF-ĸB is activated through phosphorylation and ubiquitin-dependent degradation of IĸB by CRL/SCF^βTrCP^ E3 ubiquitin ligase to turn on its downstream target genes. USP15, in association with CSN, deubiquitinates and promotes IĸB stabilization by inhibiting CRL/SCF^βTrCP^ activity and hinders NF-ĸB signaling (**left**) [33]. USP15 negatively regulates Wnt/β-catenin signaling by promoting the formation of the β-catenin destruction complex. In association with CSN, USP15 deubiquitinates and induces the stability of the essential component of the β-catenin destruction complex APC, which is required to target the phosphorylated β-catenin for CRLβ-TrCP-dependent proteasomal degradation (**right**) [35].

The transcription factor NF-ĸB plays a vital role in numerous cellular phenomena, including cell proliferation, differentiation, inflammation, and immunity. The aberrant activation of NF-ĸB signaling is involved in several diseases, but predominantly in cancer progression, thereby demanding its tight regulation in cells. In steady-state cellular conditions, NF-ĸB remains inactivated through its interaction with the inhibitor protein IĸB in the cytosol. However, in response to multiple stimuli, IĸB is phosphorylated by IĸB kinase (IKK) on Ser32/Ser36 residues, leading to its ubiquitination and subsequent proteasomal degradation induced by CRL/SCF^βTrCP^ E3 ubiquitin ligase, resulting in the release of active NF-ĸB, translocation to the nucleus, and expression of its downstream target genes [64]. Conversely, IĸB is stabilized by inhibition of the CRL/SCF^βTrCP^ activity, which prevents its ubiquitin-dependent degradation through deubiquitination by USP15 in association with CSN. Thus, USP15 inhibits NF-ĸB signaling and its downstream target genes by deubiquitination and stabilization of IĸB in mammalian cells [33], as illustrated in Figure 2 (left). Besides, the fungal paralog of βTrCP, the F-box23 protein, promotes the ubiquitination and subsequent degradation of IĸB [65], which is protected through deubiquitination by the corresponding USP15 isoform in *A. nidulans*, UspA [59]. In this pathway, UspA decreases the NF-ĸB-like velvet domain protein VeA by stabilization of F-box23 and coordinates fungal differentiation and secondary metabolism [59]. Therefore, CSN-associated USP15 plays a critical role in the deubiquitination, stabilization, and reaccumulation of IĸB and contributes to the suppression of NF-ĸB signaling in multiple species [33,66].

Apart from CSN-mediated regulation, a recent report showed that USP15 potentiates NF-ĸB activation by inhibiting the proteolysis of TGF-β-activated kinase-1 (TAK1)-binding proteins TAB2 and TAB3. The enhanced interaction of USP15 with the TAB protein complex upon TNFα stimulation induces the cleavage of Lys48-linked ubiquitination of TAB2, resulting in TAB2 stabilization. USP15 also stabilizes TAB3 by inhibiting the autophagy cargo receptor 1 (NBR1)-mediated TAB3 autophagic degradation. Additionally, USP15 inhibits lysosomal degradation of both TAB2 and TAB3 independently of its deubiquitinase activity, resulting in enhanced TNFα- and IL-1β-induced NF-ĸB activation [67]. USP15 is also reported to activate NF-κB signaling by deubiquitination and stabilization of the transcription factor NF-κB p65, which inhibits apoptosis and induces cell proliferation in multiple myeloma [68]. Therefore, USP15 plays a dual role in both the suppression and activation of NF-κB signaling, depending on the cellular context and regulatory mechanism. Unlike USP15, the paralog USP4 has been more commonly reported as a suppressor of NF-κB signaling by the downregulation of TNFα-induced NF-κB activation via targeting of upstream regulatory proteins, such as TAK1 [69], TRAF2, and TRAF6 [52], as well as HDAC2 [70]. Another USP15 paralog, USP11, also negatively regulates TNFα-induced NF-κB activation by constitutively associating with IκBα and preventing IκBα degradation [71]. Nevertheless, additional studies are required to verify whether USP15 can directly target the components of the NF-κB complex to regulate its activation and to explore the underlying molecular mechanisms.

### 3.5. USP15 Shows Both Positive and Negative Effects on Wnt/β-Catenin Signaling

The Wnt/β-catenin signaling pathway plays a central role in cell fate determination, proliferation, migration, angiogenesis, development, and organogenesis during embryonic development. The abnormal accumulation of β-catenin and its targeted gene expression is the main driver of cellular transformation in several human malignancies [72]. In normal cellular conditions, cytoplasmic β-catenin undergoes ubiquitin–proteasome system-mediated degradation by the β-catenin destruction complex in cooperation with CSN. The formation of CSN-based supercomplexes and the CSN-mediated stabilization of the tumor suppressor protein adenomatous polyposis coli (APC) are both required for efficient β-catenin degradation. In this pathway, USP15, in association with CSN, promotes APC stabilization, the essential component of the β-catenin destruction complex, and thus negatively regulates Wnt/β-catenin signaling [35], as shown in Figure 2 (right). Further, mass spectrometric analysis of the protein-binding network in glioblastoma cells identified USP15 as an interacting partner that is homologous to the E6-AP carboxyl terminus (HECT) domain-containing E3 ubiquitin protein ligase 1 HECTD1, whose mouse homolog has been associated with an inhibitory effect on the Wnt pathway. In this system, USP15 diminishes canonical Wnt activity by deubiquitinating and stabilizing HECTD1 in a β-catenin-dependent manner, suggesting a tumor-suppressing role of USP15 in glioblastoma [73]. In contrast, during bone formation by osteoblasts, USP15 prevents ubiquitin-dependent proteasomal degradation of β-catenin, thereby activating an alternative pathway of Wnt signaling. In this process, mitogen-activated protein kinase MEKK2-mediated phosphorylation of β-catenin at serine 675 promotes the recruitment of USP15, which, in turn, enhances β-catenin stability and osteoblast differentiation [74]. Therefore, USP15 can either suppress or enhance Wnt signaling, depending on its regulatory role in the degradation or stabilization of target proteins. USP4 is also identified as a β-catenin-specific DUB that most likely acts as a positive regulator of Wnt/β-catenin signaling in colorectal cancer by promoting β-catenin-regulated gene transcription [75]. However, to date, there are no reports of USP11 regulating Wnt/β-catenin signaling.

### 3.6. USP15 Regulates p53 Signaling by Controlling the Stability of MDM2 and p53

The tumor suppressor protein p53, which has a very short half-life, is stimulated by a variety of cellular stress signals, resulting in its increased half-life and transcriptional activity. The protein p53 undergoes ubiquitin-dependent proteasomal degradation and functional inactivation induced by the E3 ubiquitin ligase, for example, MDM2, and E6AP, which are themselves ubiquitinated and degraded by the proteasome. USP15 deubiquitinates the E3 ligase MDM2 in colon cancer [41] and malignant hematopoiesis [76], and E6AP in cervical cancer [77,78], which prevents their ubiquitin-dependent degradation, resulting in the concomitant downregulation of p53 and target gene expression, thereby enhancing tumor cell survival. In a recent report, USP15 directly deubiquitinates p53-R175H mutant protein, which is degraded by the lysosomal pathway in ovarian cancer that finally contributes to cancer progression [79]. USP15-mediated MDM2 stabilization also negatively regulates T-cell activation through the targeted degradation of the transcription factor NFATc2 in melanoma and colorectal cancer cell lines. Thus, USP15-mediate downregulation of p53 and consequent cancer cell survival suggests that the targeted inhibition of USP15 could enhance tumor cell apoptosis and boost antitumor T-cell responses [34]. A recent study found that the TRAF-interacting protein with a forkhead-associated domain B (TIFAB), which is a signaling protein in hematopoietic and immune cells, binds to and increases the catalytic activity of USP15, resulting in the enhanced deubiquitination and stabilization of MDM2 and consequent inhibition of the tumor-suppressive activity of p53 [80]. An additional report showed that the USP15-dependent lysosomal pathway regulates the turnover of the gain-of-function mutant of p53, p53-R175H, wherein USP15 acts as a selective upstream regulator to stabilize the mutant p53 protein. Depletion of USP15 causes decreased cell viability of ovarian cancer cells expressing the p53-R175H mutant protein, suggesting that targeting USP15 in p53-R175H mutant-containing tumors would facilitate the inhibition of oncogenic mutant p53 and enhance cancer immunotherapy [81]. Furthermore, USP15 stabilizes the oncoprotein E6, which interferes with p53 tumor suppressor activity. The protein p53 interacts with E6 through the E3 ubiquitin ligase E6AP to form a complex that ubiquitinates and targets p53 for ubiquitin-dependent proteasomal degradation. At this point, USP15 interacts with and stabilizes E6 protein levels by deubiquitinating and inhibiting its proteasomal degradation. However, while siRNA-mediated knockdown of *USP15* led to a reduction in E6 protein levels, the overall p53 levels did not increase, which indicates that additional compensatory mechanisms are involved in the modulation of p53 levels. Probably, the siRNA-mediated reduction in E6 levels may not be sufficient to influence cellular E6/E6AP activity for the ubiquitination and degradation of p53 [82]. Alternatively, other major regulators of p53 in HPV-positive cells, such as MDM2, COP1, and Pirh2, may compensate to balance the effects of the lowered p53 levels [83], which needs further research to clarify this point.

**Figure 3:**
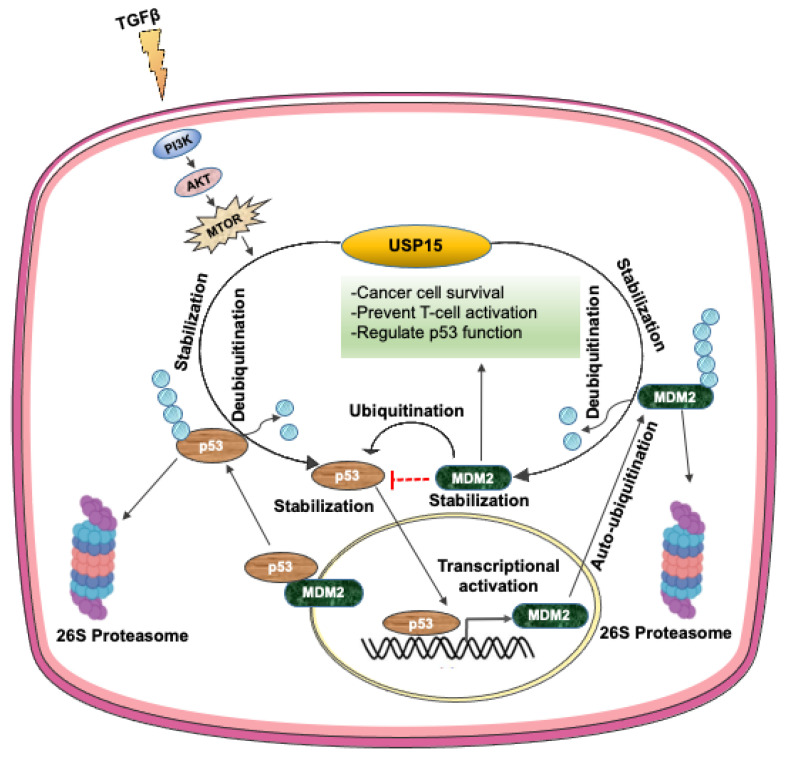
Regulation of p53 signaling by USP15. USP15 translation is upregulated by TGF-β through the PI3K/AKT pathway, which in turn increases the stabilization of p53 by its deubiquitination and activates p53 signaling. In another way, USP15 also promotes the ubiquitin-dependent degradation of p53 by deubiquitination and enhanced stabilization of the E3 ubiquitin ligase for p53, MDM2, resulting in the downregulation of p53 target gene expression [34,84].

A recent study found that USP15 translation is upregulated by TGF-β through the PI3K/AKT pathway, which, in turn, increases the stabilization of p53 by its deubiquitination in U2OS and HEK293 cells. Briefly, p53 stability is maintained by TGF-β signaling through complex formation with the TGF-β component SMAD2/3, which activates the transcription of a variety of tumor suppressor genes, including p53. In this pathway, USP15 binds to and promotes the stabilization of p53 by deubiquitination. USP15 translation is promoted by TGF-β via the activation of the mammalian target of rapamycin mediated by the PI3K/AKT pathway [84]. Thus, USP15 exerts both suppressing and inducing effects on p53 signaling, either by promoting the degradation of p53 or by stabilizing p53, respectively (Figure 3), which, in turn, regulates cancer cell progression. Nevertheless, the paralog USP4 probably suppresses p53 signaling by deubiquitination and stabilization of p53-targeting E3 ligases, which are different from those targeting USP15, such as ARF-BP1 [85] or HDAC2 [70]. Thus, USP4 predominantly acts as a tumor promoter by inhibiting p53 transcription and proapoptotic function. In contrast, USP11 is more likely to act as a positive regulator of p53 activation by deubiquitinating and stabilizing p53 in response to DNA damage [86] or by regulating IKKα-mediated p53 stabilization upon exposure to TNFα [76].

### 3.7. USP15 Attenuates IGF Signaling in Prostate Cancer

Insulin-like growth factor (IGF) signaling is mediated by the insulin receptor substrate (IRS)-1/2 through phosphorylation by IGF-I receptor tyrosine kinase upon ligand binding that promotes the survival, proliferation, and differentiation of several cell types [77,79]. IGF signaling is induced by enhanced tyrosine phosphorylation of IRS-2, which is facilitated by the E3 ligase Nedd4-dependent monoubiquitination of IRS-2. Nedd4-mediated IRS-2 ubiquitination is inhibited by USP15 to attenuate downstream IGF-I signaling in prostate cancer PC-3 cells [87]. As excessive IGF activity is reported to promote cancer progression [88], the induction or suppression of USP15-mediated IRS-2 deubiquitination by selective hormones may enable the fine-tuning of IGF-induced growth-promoting activity. Unlike USP15, USP4 is not directly involved in the regulation of IGF signaling. However, USP4 is phosphorylated upon IGF-I and TGF-β induction, and depletion of USP4 reduces IGF-I-Myr-AKT-induced migration in MDA-MB-231 breast cancer cells [31]. Furthermore, USP11 suppresses IGF-I- or EGF-induced activation of the PI3K/AKT pathway by deubiquitination and stabilization of the tumor suppressor PTEN. However, IGF stimulation triggers AKT phosphorylation in USP11-depleted cells, resulting in tumor progression, which suggests a critical role of USP11 in the IGF-induced signaling network [89].

### 3.8. USP15 Promotes RIG-I-Mediated Antiviral Innate Immune Signaling

Regulation of the immune signaling pathways by reversible ubiquitination is critical for proper innate immune responses against viral infections. The E3 ligase tripartite motif protein 25 (TRIM25)-mediated attachment of Lys63-linked ubiquitin chains activates the viral sensor, a retinoic acid-inducible gene I (RIG-I), resulting in the stimulation of the production of the cytokines IFN-α and IFN-β upon viral infection. However, TRIM25 and RIG-I are independently targeted by the linear ubiquitin assembly complex (LUBAC), consisting of two RING-IBR-RING (RBR)-containing E3 ligases, HOIL-1L and HOIP, and efficiently suppress the production of virus-induced IFN. The LUBAC complex mediates linear ubiquitination and enables the formation of K48 chains on TRIM25 to induce its proteasomal degradation, thereby inhibiting the RIG-I signaling pathway and suppressing virus-induced IFN production [90]. Protein purification and subsequent mass spectrometric analysis identified USP15 as a TRIM25-interacting protein that deubiquitinates and stabilizes TRIM25 by preventing LUBAC-dependent degradation and enhancing type I IFN production [91]. Thus, RIG-I- and TRIM25-mediated rapid production of type I IFN is facilitated by USP15 to fight against virus infection. However, effective control of antiviral signaling is also necessary to prevent excessive IFN production, which may cause autoimmune diseases. Therefore, a thorough understanding of the molecular mechanisms and fine balancing of immune signaling is necessary to control infectious diseases and to develop therapeutic targets for autoimmune disorders. USP15 might be a potential candidate as a critical regulator of antiviral innate immune signaling by limiting IFN production. Like USP15, USP4 plays critical roles in immune homeostasis, albeit via different cellular mechanisms, such as the regulation of TLR/IL-1R signaling through cleavage of the Lys63-linked polyubiquitin chain on TRAF6 [92] or the stabilization and promotion of the function of interferon regulatory factor 8 (IRF8) [93] and IRF4 [94]. USP11 also exerts a noncatalytic function to negatively regulate TNFα-mediated NF-κB activation, which may modulate multiple signaling pathways with a wide range of downstream effects on immune responses [71].

### 3.9. USP15 Downregulates Nrf2 Signaling and Target Gene Expression

Nuclear factor erythroid 2-related factor (Nrf2), a master regulator of the antioxidant response, undergoes proteasomal degradation after polyubiquitination by the Keap1–Cul3–E3 ligase complex. In response to the inducers, USP15 deubiquitinates the substrate adaptor protein of the Cul3-dependent E3 ligase complex Keap1. Keap1 deubiquitination promotes its incorporation to the Keap1–Cul3–E3 ligase complex and enhances the complex stability and enzymatic activity, which, in turn, increases Nrf2 protein degradation, resulting in reduced Nrf2 signaling and target gene expression. Moreover, the depletion of USP15 causes paclitaxel resistance through the upregulation of Nrf2 [38,95]. Recently, USP15 inhibition was found to prevent glutamate-induced oxidative damage and neurotoxicity by activating the Nrf2/HO-1 signaling pathway in HT22 cells [96], suggesting that USP15 might be a promising therapeutic target to prevent disease progression associated with Nrf2 activation. Such regulation of Nrf signaling by USP4 and USP11 has not yet been reported to date.

The above-described USP15-regulated signaling pathways with their target substrates and subsequent cellular effects are summarized in Table 1.

## 4. Coupling of Signal Transduction to Pre-mRNA Splicing Regulated by USP15

Alternative pre-mRNA splicing is a fundamental cellular mechanism that gives rise to multiple functionally distinct proteins from a single gene. Coupling of mRNA splicing to extracellular signaling is critical to establish splicing patterns during cell differentiation and development according to the physiological state of cells. The recognition of signal-induced changes in alternative splicing has been rapidly growing in the past few years, particularly concerning the mechanisms by which the activities of the splicing regulatory proteins are regulated by different signal transduction pathways. These new mechanistic paradigms have highlighted several pathways, including regulation of the splicing machinery or the spliceosomes [97].

Besides regulating several signaling pathways discussed here, USP15 is also involved in the core post-translational modifications that rearrange spliceosome components. Along with its substrate targeting factor SART3, USP15 deubiquitinates the U4/U6 spliceosomal component PRP31 following modification with Lys63-linked ubiquitin chains by the PRP19 complex. In this process, USP15 makes a complex with USP4 and SART3 that serves as a platform for the efficient splicing of chromosome segregation-related genes, such as Bub1 and α-tubulin, by stabilizing the U4/U6.U5 tri-snRNP components. Therefore, USP15 and USP4 play vital roles in the regulation of dynamic protein–protein interactions of the spliceosome. The misregulation of this process causes defects in the splicing of chromosome segregation-related genes, resulting in aberrant cell cycle progression [24,98]. USP15 and USP4 also undergo phosphorylation, and this phosphorylated status is critical for their localization in the nucleus, which is required to regulate the spliceosome dynamics during mRNA splicing [25]. There is evidence that aberrant mRNA splicing of the components of the NF-κB signaling pathway contributes to tumorigenesis and other human diseases [99]. Therefore, USP15-mediated gene splicing may also activate downstream signaling networks. Moreover, the related signaling pathways can, in turn, influence the splicing machinery, although this has not yet been investigated in detail. Comprehensive knowledge from additional studies of how extracellular stimuli are communicated to specific proteins and regulate spliceosomal activity is necessary to address the signal-responsive splicing regulation by USP15. Hence, a better understanding of the cellular concentrations and mechanisms of the core splicing machinery in the regulation of disease-associated signal transduction pathways may reveal a group of novel therapeutic targets.

## 5. Misregulation of USP15 in Diseases

Inappropriate regulation of USP15 modification is associated with certain types of cancer and several other diseases [100,101,102,103]. USP15 exhibits an oncogenic role in cervical cancer by promoting the stabilization of HPV oncoproteins [82]. Additionally, USP15 expression is correlated with paclitaxel sensitivity in ovarian cancer [104] and docetaxel sensitivity in gastric cancer [105], whereas elevated USP15 expression in human glioblastoma is found to be correlated with a shorter life expectancy [13]. Moreover, the Cancer Genome Atlas showed deletion of USP15 in 16% of breast cancers and 5% of pancreatic cancers. Furthermore, *USP15* mutations can increase the sensitivity of cancer cells to poly ADP ribose polymerase (PARP) inhibitors that selectively kill breast and ovarian cancer cells with defects in homologous recombination. Therefore, USP15 may act as a potential biomarker for predicting the responses of pancreatic, breast, and ovarian cancers to various treatments [106]. Besides its oncogenic role, USP15 also antagonizes the E3 ubiquitin ligase Parkin-mediated mitochondrial ubiquitination and inhibits selective autophagy of damaged mitochondria (mitophagy) in Parkinson’s disease (PD) [107]. In addition, a microarray analysis approach identified decreased USP15 expression in the neurodegenerative disorder spinocerebellar ataxia type 3 (also known as Machado–Joseph disease) and in Huntington’s disease [108]. A recent study revealed the role of USP15 in epilepsy pathogenesis, where *USP15* silencing protects against glutamate-mediated neurotoxicity. These findings suggest that pharmacological inhibition of USP15 may provide a novel antiepileptic target against epilepsy or other oxytocin-associated neurological disorders [96]. Additionally, USP15 regulates antiviral innate immune responses by deubiquitinating and stabilizing the ubiquitin ligase TRIM25, resulting in the activation of a type I interferon response that exacerbates microbial responses and autoimmune neuroinflammation [91,109]. However, the dual role of USP15 in suppressing or activating a particular signaling pathway highlights its complex and critical regulatory roles, depending on the different cellular contexts. The findings of several recent reports suggest that deubiquitinating enzymes may be considered cancer biomarkers or predictors of cancer drug resistance, which can provide new therapeutic opportunities and guide the design of gene therapies or inhibitors to improve cancer diagnosis and prognosis [110,111]. Therefore, further research based on the current evidence will lead to a better understanding of the role of USP15 in cancer and other diseases and may identify USP15 as a potential target for future treatment strategies.

## 6. Perspective

Here, we have discussed the signaling networks regulated by USP15 that are predominantly involved in various cellular pathways associated with cancer and other related diseases. From the above discussion, it is quite clear that USP15 has a wide range of target substrates that regulate a variety of signaling pathways associated with several human disorders. Evidence shows the oncogenic role of USP15 in abolishing tumor suppressor activity, such as that of p53, through deubiquitination and stabilization of oncoproteins, MDM2, and HPV type-16 E6 [34,82]. However, several researchers also found USP15 to be a negative regulator of oncogenic NF-ĸB, Wnt, or IĸB signaling [33,35]. Therefore, the molecular mechanisms underlying the specificity of USP15 for different substrates in different cellular pathways need to be further explored. Despite the insufficiency in full understanding of USP15 biology and the subsequent difficulties in designing the selective USP15 inhibitors [103], current progress in the development of potent and selective DUB inhibitors, such as mitoxantrone [112] and ubiquitin variants (UbVs) targeting USP15 catalytic domain [113], is increasing rapidly. So far, the preliminary analysis of UbVs is quite promising for USP15 inhibition with improved potency and specificity suggesting it to be a good candidate for future drug development. Based on this evidence, USP15 may provide an imperative tool for the design of new drugs based on the structure, substrate recognition, and variances among other closely related USP paralogs. Additionally, comprehensive proteomic approaches may facilitate the identification of the substrate proteins that are targeted and recognized by USP15, which, in turn, will help to understand the disease-causing mechanism of USP15. Besides, better knowledge of USP15 regulation and the expression levels in different tumor tissues will provide a concrete understanding of its role in different pathological and physiological processes, especially in cancers. Therefore, given the detailed regulatory mechanism of USP15 in a variety of diseases, the development of USP15-targeting drugs or small-molecule inhibitors might be a far-reaching and valuable goal for future research.

## Figures and Tables

**Table 1:** USP15-regulated signaling pathways, target proteins, and their effects on cells.

Pathway	Target Substrate	Mechanism of Action	Effect on Cells	Reference
TGF-β signaling	ALK5	Deubiquitination and stabilization of TβR-I by making a complex with SMAD7 and SMURF2	Enhances the activity of TGF-β signaling and promotes oncogenesis in glioblastoma	[13]
BMP signaling	ALK3	Deubiquitination of type I BMP receptor ALK3 by interacting with BMP inhibitor SMAD6	Enhances BMP target gene transcription in mammalian cells, osteoblastic differentiation in mouse, and embryogenesis in *Xenopus*	[39]
COP9 signalosome	CRLs	Deubiquitination and stabilization of CRL adaptor proteins and associated substrates	Regulation of cullin activity	[58]
			Regulation of multicellular development and secondary metabolism	[59]
			Protects and stabilizes the components of CRLs for rearrangement and adaptation to altered cellular requirements	[60]
NF-*κ*B signaling	IκBα	Deubiquitination and stabilization of IκBα	Inhibition of NF-ĸB signaling and downstream gene expression	[33]
	NF-κB p65	Inhibition of NF-κBp65 ubiquitination	Induces cell proliferation and inhibition of cell apoptosis in multiple myeloma by activating NF-κB signaling	[68]
	TAB2 and TAB3	Stabilization of TAB2 and TAB3 by inhibiting their proteolysis	Potentiates TNFα- or IL-1β-induced NF-ĸB activation and downstream gene transcription	[67]
Wnt/β-catenin signaling	APC	Stabilization of APC in the β-catenin destruction complex	Suppresses Wnt/β-catenin signaling and target gene expression	[35]
	HECTD1	Deubiquitination and stabilization of HECTD1	Tumor suppression in a subset of glioblastoma by attenuating the canonical Wnt pathway	[73]
	β-catenin	Stabilization of β-catenin by inhibiting degradation	Osteoblast differentiation and bone formation	[74]
p53 signaling	MDM2	Deubiquitination and stabilization of MDM2	Downregulation of p53 along with its target genes and cancer cell survival	[34]
	MDM2 via TIFAB	Increases the deubiquitination and stabilization of MDM2, which is boosted by TIFAB	Suppresses p53 activity in stressed and malignant hematopoietic cells	[80]
	E6	Increases E6 protein stability	Upregulation of E6 oncoproteins may facilitate cancer progression in HPV-infected cells	[82]
	p53	Binding and stabilization of p53	TGF-β signaling-mediated stabilization of p53 through increased USP15 translation that suppresses the early stages of cancer progression	[84]
IGF signaling	IRS-2	Binding to and antagonizing of IRS-2 ubiquitination	Regulation of cancer cell progression by fine-tuning of IGF-induced growth-promoting activity	[87]
Immune signaling	TRIM25	Deubiquitination and stabilization of TRIM25	Promotes antiviral innate immune responses	[91]
Nrf2–Keap1 signaling	Keap1	Deubiquitination of Keap1 and enhancement of the Keap1–Cul3–E3 ligase complex stability	Promotes Nrf2 protein degradation and reduces the Nrf2 target gene expression	[38]
